# Native and Non-Native Plants Provide Similar Refuge to Invertebrate Prey, but Less than Artificial Plants

**DOI:** 10.1371/journal.pone.0124455

**Published:** 2015-04-17

**Authors:** Bart M. C. Grutters, Bart J. A. Pollux, Wilco C. E. P. Verberk, Elisabeth S. Bakker

**Affiliations:** 1 Department of Aquatic Ecology, Netherlands Institute of Ecology (NIOO-KNAW), Wageningen, the Netherlands; 2 Experimental Zoology Group, Department of Animal Sciences, Wageningen University, Wageningen, the Netherlands; 3 Department of Animal Ecology and Ecophysiology, Institute for Water and Wetland Research, Radboud University Nijmegen, Nijmegen, the Netherlands; Texas A&M University at Galveston, UNITED STATES

## Abstract

Non-native species introductions are widespread and can affect ecosystem functioning by altering the structure of food webs. Invading plants often modify habitat structure, which may affect the suitability of vegetation as refuge and could thus impact predator-prey dynamics. Yet little is known about how the replacement of native by non-native vegetation affects predator-prey dynamics. We hypothesize that plant refuge provisioning depends on (1) the plant’s native status, (2) plant structural complexity and morphology, (3) predator identity, and (4) prey identity, as well as that (5) structurally similar living and artificial plants provide similar refuge. We used aquatic communities as a model system and compared the refuge provided by plants to macroinvertebrates (*Daphnia pulex*, *Gammarus pulex* and damselfly larvae) in three short-term laboratory predation experiments. Plant refuge provisioning differed between plant species, but was generally similar for native (*Myriophyllum spicatum*, *Ceratophyllum demersum*, *Potamogeton perfoliatus*) and non-native plants (*Vallisneria spiralis*, *Myriophyllum heterophyllum*, *Cabomba caroliniana*). However, plant refuge provisioning to macroinvertebrate prey depended primarily on predator (mirror carp: *Cyprinus carpio carpio* and dragonfly larvae: *Anax imperator*) and prey identity, while the effects of plant structural complexity were only minor. Contrary to living plants, artificial plant analogues did improve prey survival, particularly with increasing structural complexity and shoot density. As such, plant rigidity, which was high for artificial plants and one of the living plant species evaluated in this study (*Ceratophyllum demersum*), may interact with structural complexity to play a key role in refuge provisioning to specific prey (*Gammarus pulex*). Our results demonstrate that replacement of native by structurally similar non-native vegetation is unlikely to greatly affect predator-prey dynamics. We propose that modification of predator-prey interactions through plant invasions only occurs when invading plants radically differ in growth form, density and rigidity compared to native plants.

## Introduction

Non-native species are becoming widespread due to globalization and can have a profound effect on ecosystem functioning by altering the structure of food webs [1, 2]. Commonly underlying these food web effects are changes in nutrient cycling and habitat structure [3, 4]. As predator-prey dynamics are typically mediated by the structural complexity provided by plants [5–9], ongoing plant invasions may distort predator-prey dynamics and ultimately ecosystem functioning through food web effects. Non-native plants may differentially shape the refuge provided to prey [10–12] or change prey behaviour, *i*.*e*. through differences in the structural complexity or density of native and non-native plants. Indeed, behavioural changes in prey have been induced by non-native plants in both terrestrial [13, 14] and aquatic habitats [15].

Plants can change predator and prey behaviour [16, 17] and alter predator-prey dynamics by providing physical predator-free refuge [5, 7] or by reducing encounter rates and prey visibility [8]. The extent to which such effects are manifested is mainly dictated by the habitat structure provided by plants, which is determined by the shoot density and architectural complexity. For example, densities > 350 artificial stems m^-2^ have been shown to impair prey (*Daphnia pulex*) detection and the swimming speed and predation rate of the planktivorous fish *Pseudorasbora parva* [18]. Similarly, increasing artificial plant density reduced predation rates of largemouth bass (*Micropterus salmoides*) feeding on bluegills (*Lepomis macrochirus*) [19] and real submerged plants reduced largemouth bass feeding on rainwater killifish (*Lucania parva*) [12]. Furthermore, a high plant structural complexity benefitted prey survival (*Anopheles* sp. larvae) under fish (*Nannoperca australis*) predation [6]. In general, finely dissected leaves, dense whorls and spiny leaf axils contribute to plant complexity and can reduce prey visibility and provide physical refuge [5, 20].

The provisioning and effectiveness of refuge also depends on predator and prey identity. For example, chironomid larvae were safer in complex plants under bream (*Abramis brama*) and roach (*Rutilus rutilus*) predation than under perch (*Perca fluviatilis*) predation [21]. Predator hunting mode (*e*.*g*. pursuing or ambushing prey [22]) can affect the role of habitat complexity in providing refuge to prey organisms [16]. For instance, some prey evade structured habitats if the risk of ambush predators is high, but they might enter structured habitats upon seeing predators in the open water [23, 24]. Additionally, the role of plants in predator-prey dynamics is further determined by prey characteristics such as size, activity, swimming speed, camouflage or susceptibility to allelochemicals [25, 26]. For example, predator size determines whether plant interstitial space hinders predation, while prey size determines whether prey can fit into the available interstitial space to survive predation attempts [27–29]. Another example is prey susceptibility to plant allelochemicals, as prey face the dilemma of having to endure these chemicals in the relative safety near the plant, or escape these chemicals by venturing out in the open but risk being preyed upon [30, 31].

Altogether, plant refuge provisioning is determined by multiple parameters and can be highly plant-, predator- and/or prey-specific [9]. Therefore, alterations in habitat structure through the replacement of native by non-native plant species can greatly alter food webs and ecosystem functioning [4, 32–34]. Yet, our understanding of the plant-mediated effects on predation dynamics by submerged native and non-native aquatic plants is still insufficient to predict invasion impacts [35].

In this study, we compare the refuge provided by submerged aquatic native and non-native plants to three macroinvertebrate prey species predated upon by actively hunting fish (mirror carp: *Cyprinus carpio* L. 1758) or ambushing dragonfly larvae (*Anax imperator*) in laboratory trials. We hypothesize that (1) non-native submerged macrophytes provide less refuge than native aquatic plants to macroinvertebrate prey as the unfamiliarity of native prey species with non-native plants may limit their optimal utilization of these novel sources of refuge, leaving them potentially more vulnerable to predation. In addition, we expect that (2) plant structural complexity and shoot density increase the effectiveness of refuge provisioning, and that plant refuge provisioning depends on (3) predator and (4) prey identity [9]. In addition to experiments with living macrophytes, we performed predation trials with artificial plants to exclude allelopathy and purely assess the role of plant complexity. We hypothesize (5) that artificial and living structures similar in density and complexity provide similar refuge.

## Materials and Methods

### Ethics statement

The authors declare that mirror carp feeding trials comply with the animal research laws of the Netherlands and permission for these was provided by the Royal Netherlands Academy of Arts and Sciences animal sciences committee under application NIOO 13.09.

The macroinvertebrates in this study were collected from non-protected privately owned streams or artificial ponds with permission from the landowner and the regional water board. All odonate species used are listed on the IUCN Red List as ‘of least concern’. We took care that our sampling of individuals for use in laboratory predation trials would not threaten local populations of macroinvertebrate species.

### Experimental design

We assessed the degree of refuge provided to macroinvertebrate prey species by native and non-native submerged plants by means of predation trials. Each predation trial consisted of a predator foraging on multiple individuals of a single prey species in an aquarium with one plant species being present, or without any plants (see description below). The six selected native and non-native plants are common in European waters [36] and vary in structural complexity, as expressed by their fractal dimensions [37] (Table 1). The plants were tested using two contrasting shoot densities (‘low’ vs ‘high’), except in experiments with water flea (*Daphnia pulex*). Water fleas clustered in the corners of the experimental area, *i*.*e*. away from the plants, already at low shoot density, so we did not further assess them at high shoot density. Three different experiments were performed to test plant refuge provision.

**Table 1:** Information on the real and artificial aquatic plants used.

Plant species	Origin	Wet mass (g)	Dry mass (g)	PVI (% volume)	Architecture	Fractal dimensionof cross-section (D)	Fractal dimensionof shoot (D)
*Potamogeton perfoliatus*	native	13.0	1.1	23	broad leaves	1.84 ± 0.04	1.70 ± 0.05
*Myriophyllum spicatum*	native	34.7	3.2	26	whorls of dissected leaves	1.55 ± 0.12	1.76 ± 0.03
*Ceratophyllum demersum*	native	93.6	5.6	26	dense whorls	1.58 ± 0.06	1.83 ± 0.03
*Vallisneria spiralis*	non-native	33.9	1.7	23	singular leaves in rosettes	1.37 ± 0.13	1.71 ± 0.05
*Myriophyllum heterophyllum*	non-native	35.4	2.9	26	whorls of dissected leaves	1.39 ± 0.07	1.73 ± 0.03
*Cabomba caroliniana*	non-native	40.7	2.0	26	pairs of dissected leaves	1.71 ± 0.05	1.81 ± 0.05
Vallisneria	plastic	-	-	23	singular leaves	1.34 ± 0.17	1.83 ± 0.03
Elodea	plastic	-	-	23	whorls	1.60 ± 0.02	1.74 ± 0.01
Myriophyllum	plastic	-	-	23	dissected leaves	1.35 ± 0.04	1.77 ± 0.03
Ceratophyllum	plastic	-	-	23	dense whorls	1.49 ± 0.02	1.86 ± 0.01

Overview of the aquatic plants in the predation trials along with information regarding the wet and dry weight of the native, non-native and plastic plant monocultures as well as their biomass, percent volume infested (PVI), morphological description and fractal dimension.

In the first experiment we tested the refuge provided by three native (*Potamogeton perfoliatus*, *Myriophyllum spicatum* and *Ceratophyllum demersum*) and three non-native plant species (*Vallisneria spiralis*, *Myriophyllum heterophyllum*, *Cabomba caroliniana*) to three widespread macroinvertebrate species varying in size and activity [38, 39] predated on by actively hunting juvenile mirror carp (*C*. *carpio*). As prey, we used a motile benthic amphipod (*Gammarus pulex* L. 1758), a small pelagic zooplankter (*Daphnia pulex* Leydig 1860) and sedentary phytophilic damselfly larvae (approximately 70% *Ischnura elegans* Vander Linden 1820 and 30% *Coenagrion puella* L. 1758 or *C*. *pulchellum* Vander Linden 1825).

To compare the refuge provision to prey predated by predators differing in their hunting mode [40], we performed a second experiment where we measured the refuge provided by the same three native and three non-native plant species to *G*. *pulex* under predation by ambushing dragonfly larvae (*Anax imperator* Leach 1815). Dragonfly larvae are one of the primary invertebrate top predators in waters without fish, and frequently involved in predation studies [5, 25]. *G*. *pulex* are a natural food source for dragonfly larvae [41] and served as prey. Damselfly larvae were unavailable after the carp predation trials, while *D*. *pulex* is not a major food source for *A*. *imperator* [42], therefore these prey were not tested.

Artificial plant analogues are frequently used to unravel the mechanisms involved in plant refuge provision [6, 18]. In the third experiment we tested whether refuge provision by four artificial plants of varying complexity (Table 1) to *G*. *pulex* under carp predation is equal to that provided by living plants of similar structural complexity.

### Aquatic plants

**Fig 1:**
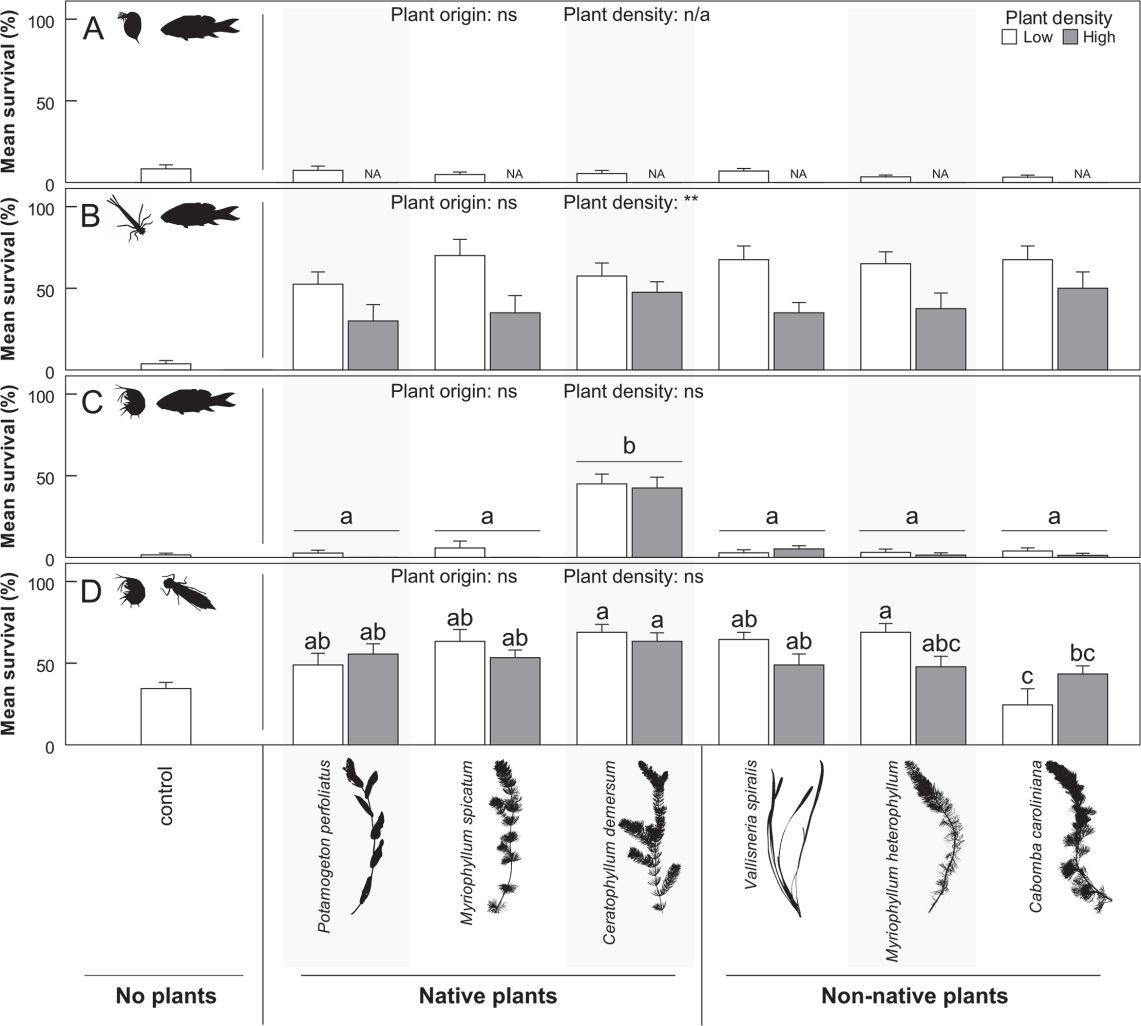
Refuge provisioning by native and non-native aquatic plants. Mean ± SEM survival (%) of (**A**) *Daphnia pulex*, (**B**) damselfly larvae and (**C**) *Gammarus pulex* under mirror carp predation (*Cyprinus carpio*; n = 8) and of (**D**) *Gammarus pulex* under *Anax imperator* predation (n = 9) in low (white bars; 300 shoots m^-2^) and high density (grey bars; 800 shoots m^-2^) plant monocultures grouped into native (left side) and non-native species (right side). Horizontal bars represent the groups that were compared. Comparisons between two groups are shown as non-significant (ns) or one to three asterisks (GLMM Wald χ^2^ tests: * *P* < 0.05; ** *P* < 0.01; *** *P* < 0.001), whereas lowercase letters indicate significance among three or more groups (GLMM simultaneous inference post hoc; *P* < 0.05). ‘NA’ indicates not available.

**Fig 2:**
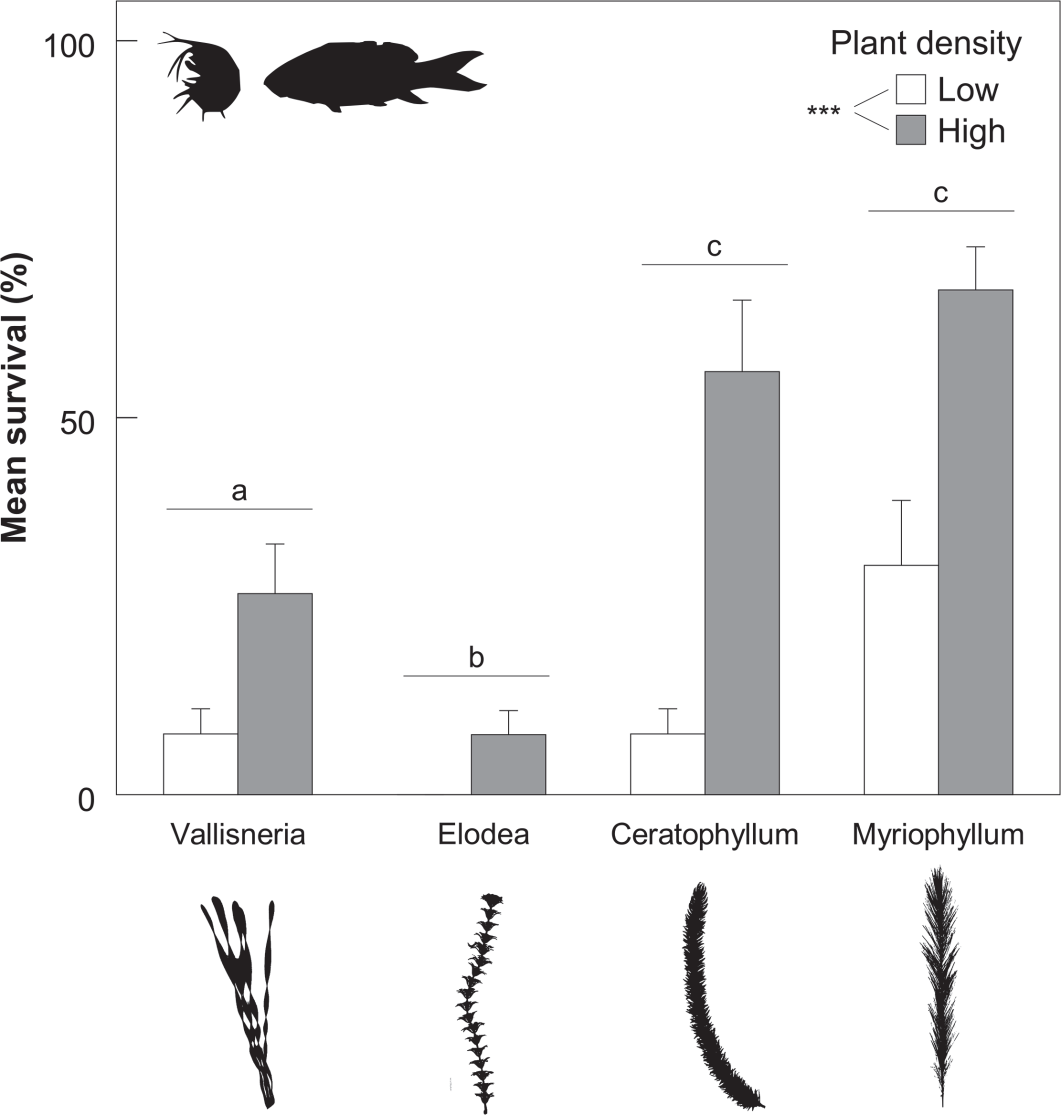
Refuge provisioning by artificial aquatic plants. Mean ± SEM survival (%; n = 8) of the benthic crustacean (*Gammarus pulex*) predated upon by mirror carp (*Cyprinus carpio carpio*) in the presence of artificial plant analogues of varying complexity and in low (white bars; 300 shoots m^-2^) and high density (grey bars; 800 shoots m^-2^). Horizontal bars indicate the groups that were compared, where comparisons between two groups are shown as either non-significant (ns) or their significance using asterisks (GLMM Wald χ^2^ tests: * *P* < 0.05; ** *P* < 0.01; *** *P* < 0.001) and lowercase letters for significance among three or more groups (GLMM simultaneous inference post hoc; *P* < 0.05).

Plants were collected from monocultures maintained in tanks located at the Netherlands Institute of Ecology (51.9879 N, 5.6724 E). We selected three common native and three non-native Northwestern European submerged plant species with similar structural complexity (visualised in Fig 1). Common, dominant species were picked as these are expected to provide most of the ecosystem functions [43]. Effectively, dominant non-natives are expected to replace dominant natives, and its effects on refuge provision are being tested. In addition to these living plant species, four artificial plant analogues resembling *Ceratophyllum*, *Myriophyllum*, *Vallisneria* and *Elodea* were purchased for use in the third experiment (Hardeman Aquarium, Ede, Netherlands; visualised in Fig 2). After harvesting, the shoots were rinsed, cut to 25 cm and had their base wrapped in foam before being attached to a metal grid in low (~ 300 shoots m^-2^) or high (~ 800 shoots m^-2^) density using binding wire (S1 Fig). These densities were based on actual shoot densities in natural plant beds [19, 44] and prior experimental work [18]. The leaves of adjacent shoots touched each other at high density. The qualitative rigidity of all artificial plant analogues, *C*. *demersum*, and *M*. *spicatum* are described using a photograph (S2 Fig). Five fresh shoots per species were scanned (Epson Perfection 4990 Photo) and analysed for their area fractal dimension at whole-shoot and cross-sectional scale using ImageJ following [37]. The fractal dimension was calculated at these two scales as each provides different information [37]. Specifically, the shoot scale encompasses whole-plant complexity of leaf width and internode length, whereas the cross-sectional fractal dimension assesses leaf-scale complexity such as the degree of leaf dissection. After completing all the predation trials, plant wet and dry mass (60°C to constant dry weight) were determined. Shoots that had turned brown were replaced with fresh specimens during the experiment.

### Macroinvertebrate prey

Damselfly larvae (14 ± 2.0 mm mean length ± SD; n = 280) were collected from two experimental ponds located at the Netherlands Institute of Ecology (51.9879 N, 5.6724 E), while *G*. *pulex* individuals (12.4 ± 2.4 mm mean length ± SD; n = 240) were collected from the Heelsumse beek (51.976 N, 5.754 E) and *D*. *pulex* individuals (1–3 mm) were commercially acquired every 4 days. All macroinvertebrate prey species were housed in aerated water of 21°C and used in predation trials after 24–72 h. *G*. *pulex* fed on dead plant material from the Heelsumse Beek while *D*. *pulex* fed on yeast and microalgae (*Scenedesmus* sp.). We photographed *G*. *pulex* and damselfly larvae to quantify their body size using ImageJ [45] and compare damselfly species survival. Visual inspection of pre- and post-trial photographs did not show differential survival for the two damselfly genera used. Damselfly larvae survivors were re-used in equal numbers across treatments (200 out of 640 individuals used) as not enough individuals could be collected from the field. The survival probability of fresh versus re-used damselfly larvae was similar (respectively 44 ± 5% versus 48 ± 5% survival; mean percent survival ± SD; calculated across all feeding trials involving damselfly larvae).

### Carp trials

In the first and third experiment we used sixteen juvenile mirror carp (5 cm) acquired from the Aquatic Research Facilities of Wageningen University and Research Centre (ARF-WUR). These were paired based on their wet mass to reduce stress and variance in predation rate, yielding 8 homogenous pairs (mean ± SD weight of 20.6 ± 0.1 g), which we randomly allocated to one of 8 aquaria on the 30th of August 2013. We fed fish with sinking pellets (Trouvit, Trouw & Co., Putten, the Netherlands; 1% wet mass day^-1^) for a week and then started to supplement their diet with living *Gammarus pulex* and later zooplankton and damselfly larvae in the three weeks prior to the trials.

The carp trials were performed at a temperature of 21°C in eight glass aquaria (180 x 40 x 40 cm in length x width x height; water depth of 38 cm; 274 L). Pairs of aquaria were connected to four biological filters. Water was added weekly to compensate for evaporation, while 50% water replenishments took place in between trials. Each aquarium was divided into three compartments using separators consisting of a wooden frame covered with 0.5 mm PE mesh (S1 Fig). In this way we constructed a fish living area (60 cm of total length), an experimental area (40 cm) and a plant storage area (80 cm) in each aquarium. Fish could swim from their living area to the experimental area through a hatch, so there was no need of transferring them with a net. This is important because the manual transfer of fish by means of hand nets would cause severe stress, affecting fish prey capture behavior for prolonged periods of time. Therefore, during the two weeks prior to the trials, we accustomed the fish to swimming through the hatch into the experimental area. Both the living and experimental areas were covered in white paper on the outside to exclude effects of external stimuli during predation trials. Artificial plants, distinctly different from all other plants, were added to the living compartment as cage enrichment so that carp could hide if desired, whereas a 4 cm layer of coarse sand was added to the experimental area. Before each predation trial, plant grids of the appropriate species were buried in the sediment in such a way that a 10 cm perimeter of open water remained around the plants. Illumination was provided by dimmable fluorescent lamps (Philips MASTER TL5 HE 28W/840) and provided 14 ± 2.1 lux (mean ± SD) measured 5 cm above the sediment in the experimental area.

All carp trials were performed within five weeks, first using real plants then with artificial plant analogues. Two (living plants) to four trials (artificial plants) a day were performed over a consecutive period of at most 8 days for each predator-prey combination according to a randomized block scheme (n = 8). As training effects may have occurred over time, we included time as a random effect (see Data analysis). In the trials, prey were acclimated to the experimental area for 10 minutes before we let the fish enter the experimental area through the hatch. As *G*. *pulex* tended to ‘escape’ into the fish living area upon opening the hatch, they were added after the fish. The carp were allowed to forage on fifty *D*. *pulex* for 10 minutes and for 30 minutes on ten *G*. *pulex* or five damselfly larvae before re-entering their living area. These prey densities reflect the natural abundance of macroinvertebrate taxa [9]. Foraging times were based on pilot experiments to estimate the time required by predators to finish most, but not all, of the prey in vegetated patches. At the end of each trial all remaining prey were counted to obtain an estimate of survival probability. We measured the standard length, gape width and wet mass for each individual fish after completing all carp feeding trials (S2 Table).

### Dragonfly larvae trials

For the second experiment, we collected 15 *Anax imperator* larvae (body length of 39.3 ± 2.1 mm) from experimental ponds located at the Netherlands Institute of Ecology (51.9879 N, 5.6724 E). In the week prior to the trials, these ambush predators were housed individually in 2 L plastic containers containing *Elodea nuttalli* and fed a single gammarid daily (approximately 10 mm). Before each trial, the *A*. *imperator* larvae were individually transferred to separate plastic containers (41 x 30 x 24 cm) filled with 25 litres of water and 4 cm of coarse sand as sediment, according to an incomplete randomized block design (n = 9) for two or three trials a day. After 30 minutes, ten *G*. *pulex* individuals (9.1 ± 2.1 mm mean ± SD; n = 320) were added to each container. Then, we allowed the *A*. *imperator* to forage for 60 minutes before returning them to their housing. At the end of each trial all remaining *G*. *pulex* were counted to obtain survival numbers. The foraging time was adjusted to match the lower feeding rate of dragonfly larvae compared to carp. Six plant species monocultures (in low and high shoot density plus a no-plant control) were used, similarly as in the carp trials (see Carp trials; Table 1).

### Data analysis

The survival data were separately analysed for each predator-prey combination with generalized linear mixed models (GLMM) of the binomial family and fitted using Laplace approximation [46]. In all GLMMs we added random intercepts for the following random effects: ‘individuals’ (fish pairs or dragonfly individuals), day and time of day. *Gammarus pulex* survival under carp predation was corrected for escapes through the fish hatch (95 out of the 1840 individuals). Six outliers (> 2.2 interquartile range) in the *Daphnia pulex* x carp dataset were removed. These outliers were present no more than once per pair of fish (n = 8) and most of them (3 out of 6) occurred on day 1 of the *D*. *pulex* x carp trials. In these cases, daphnid survival was higher than average, likely because fish were less actively searching for prey or could not find daphnids in the corners.

For every predator-prey combination, we first fitted a GLMM to test for a difference between the plant treatments and the no-plant controls. If prey survival differed between plants and controls, the controls were excluded from the dataset to analyse the crossed fixed effects of plant species and density in a GLMM. Subsequently, to compare native and non-native plants, a separate GLMM with plant origin as the fixed effect and an additional random intercept for plant species was fitted on the dataset without controls. Hypotheses were tested by analysing fixed effects with Likelihood Ratio Tests (LRT). Post hoc comparisons of significant fixed effects were performed by simultaneous inference using Tukey contrasts. There was no overdispersion in the GLMMs as the sum of squared residuals divided by the residual degrees of freedom was approximately equal to 1, except for the model fitted on *Daphnia pulex* survival. Therefore, we resorted to the glmmPQL function in R to compare plants and controls using the aforementioned random effects structure.

Fractal dimensions of shoot and cross-section were compared among real and artificial plants, as well as between native and non-native species using 1-way ANOVAs. Residuals were normally distributed, yet some data violated the assumption of homogeneity of variances for which we used Welch’s ANOVA and a Games Howell post hoc test. Additionally, Pearson correlation and linear regression were used to correlate the shoot (D between 1.63–1.88) to the cross-sectional fractal dimension (D between 1.15–1.89) and to analyse the relation between the mean prey survival (%) and fractal dimensions respectively.

Statistics were performed using R version 3.0.3 [47] and the packages ‘lme4’ [48], ‘multcomp’ [49], ‘MASS’ [50] and ‘car’ [51].

## Results

### Plant refuge for prey under carp predation

The survival of the three macroinvertebrate prey under carp predation did not differ significantly between native and non-native plants (Fig 1; Table 2). The presence of certain plant species increased the survival of *Gammarus pulex* (χ^2^_df = 1_ = 14.1; p < 0.001) and damselfly larvae (χ^2^_df = 1_ = 83.6; p < 0.001), but not of *Daphnia pulex* (t_df = 1_ = -1.63; p = 0.11), compared to no-plant controls (Table 2; Fig 1). Observations indicated that *D*. *pulex* individuals were clustered in the corners of the experimental area, *i*.*e*. away from the plants. The avoidance of plants by *D*. *pulex* was confirmed in separate tests using artificial plant analogues. Surprisingly, only *Ceratophyllum demersum* improved gammarid survival (44% survival on average) compared to other plant species (maximum 4% survival on average) and this effect was found regardless of plant shoot density (Fig 1C; Table 2). Observations revealed that *G*. *pulex* individuals settled on plants, but were then often detected and hunted down by the carp. Survival of damselfly larvae was similar among plant species (χ^2^_df = 5_ = 5.57; p = 0.35), though damselfly larvae survival was reduced at high compared to low plant density (respectively 34% and 55% average survival; Fig 1B; χ^2^_df = 1_ = 5.75; p < 0.01).

**Table 2:** Output of generalized linear mixed models (GLMM) on prey survival data.

		Mirror carp predation									Dragonfly larvae predation		
		*Daphnia pulex*			Damselfly larvae			*Gammarus pulex*			*Gammarus pulex*		
	Plants versus control	p = 0.11			**p < 0.001**			**p < 0.001**			**p < 0.001**		
GLMM for species identity and density	Fixed effects	χ^2^	df	p-value	χ^2^	df	p-value	χ^2^	df	p-value	χ^2^	df	p-value
												
	Plant species	-	-	-	5.57	5	0.35	189.4	5	**< 0.001**	45.37	5	**< 0.001**
	Density	-	-	-	5.75	1	**0.017**	0.88	1	0.35	2.20	1	0.14
	Species * Density	-	-	-	3.80	5	0.58	9.53	5	**0.090**	21.75	5	**< 0.001**
												
	Random effects	Variance	SD		Variance	SD		Variance	SD		Variance	SD	
												
	Day	-	-	-	0.094	0.31		0.14	0.38		0.24	0.49	
	Time of day	-	-	-	< 0.001	< 0.001		< 0.001	< 0.001		0.088	0.30	
	Individual	-	-	-	0.042	0.20		0.098	0.31		< 0.001	< 0.001	
GLMM for origin	Fixed effects	χ^2^	df	p-value	χ^2^	df	p-value	χ^2^	df		χ^2^	df	p-value
												
	Origin	-	-	-	1.29	1	0.26	0.84	1	0.36	1.29	1	0.26
												
	Random effects	Variance	SD		Variance	SD		Variance	SD		Variance	SD	
												
	Day	-	-	-	0.40	0.63		0.24	0.49		0.24	0.49	
	Time of day	-	-	-	< 0.001	< 0.001		< 0.001	< 0.001		0.084	0.29	
	Individual	-	-	-	0.041	0.020		0.13	0.36		0.007	0.086	
	Plant species	-	-	-	< 0.001	< 0.001		1.72	1.31		0.14	0.38	

Each predator-prey combination was modelled separately due to the large number of random effects. Origin denotes the comparison in refuge provisioning of native and non-native plant species.

### Structural complexity

Even though prey survival was largely similar across plant species, there were clear differences in structural complexity among plant species (Table 1), whereas complexity was similar for native (mean ± SD of respectively shoot and cross-sectional D: 1.76 ± 0.07 and 1.66 ± 0.16) and non-native plant species (mean ± SD of shoot D: 1.75 ± 0.05 and cross-sectional D: 1.49 ± 0.19; One-way ANOVAs for shoot complexity: F_1,4_ = 0.045, p = 0.84 and cross-sectional complexity: F_1,4_ = 1.34, p = 0.31). Both the shoot and cross-sectional fractal dimension varied among plant species (One-way Welch’s ANOVAs of respectively F_5, 11.04_ = 6.54, p = 0.005 and F_5, 38.4_ = 117.28, p < 0.001; Table 1). A high shoot complexity was not necessarily coupled to a high cross-sectional complexity (Pearson correlation, r = -0.16, n = 10, p = 0.66). Linear regression indicated a significant positive relationship between the damselfly larvae survival under carp predation and shoot fractal dimension in the treatment with high shoot density (R^2^ = 0.81, n = 6; p = 0.009) while all other tested relationships between living plant structural complexity and prey survival were non-significant (S3 Fig).

### Predation by ambushing dragonfly larvae

Under *Anax imperator* predation, *G*. *pulex* survival was higher in the presence of plants (mean ± SD survival of 53 ± 19%) compared to the no-plant control (mean ± SD survival of 34 ± 11%; χ^2^_df = 1_ = 13.6; p < 0.001; Fig 1D). Native and non-native plants provided equal refuge to *G*. *pulex* (respective mean ± SD survival of 59 ± 18% versus 48 ± 22%) and there was no effect of plant density (χ^2^_df = 1_ = 2.20; p = 0.14; Table 2). Yet there was a significant interaction of species and density (χ^2^_df = 5_ = 21.8; p < 0.001) as the survival of *G*. *pulex* in low density monocultures of *Cabomba caroliniana* had a decreased survival compared to the other plant species (Fig 1D).

### Refuge provision by artificial plants

Artificial plant analogues differed in their shoot and cross-sectional fractal dimension (1-way Welch’s ANOVAs of respectively F_3, 7.78_ = 578.3, p < 0.001 and F_3, 4.17_ = 36.6, p = 0.002; Table 1). The survival of *G*. *pulex* under carp predation was affected by artificial plant type (χ^2^_df = 3_ = 115.6; p < 0.001). Specifically, average *G*. *pulex* survival was highest in *Ceratophyllum* (32%) and *Myriophyllum* artificial analogues (49%), somewhat lower in *Vallisneria* (17%) and lowest in *Elodea* analogues (4%; Fig 2). Yet, there was no relationship between *G*. *pulex* survival and the artificial plant fractal dimensions (S3 Fig). Lastly, at high shoot density *G*. *pulex* survival increased about three-fold compared to a low shoot density (average survival of respectively 39% versus 12%; χ^2^_df = 1_ = 49.23; p < 0.001). Observations indicated that at high shoot density, fish penetrated vegetation less often, or in the case of *Ceratophyllum* and *Myriophyllum* analogues, they rarely did so. Like in living *Ceratophyllum demersum* plants, surviving gammarids in artificial plant analogues were well hidden in interstitial spaces and it took considerable effort to remove them.

## Discussion

We found that native and non-native aquatic plants generally provided equal refuge to macroinvertebrate prey. However, refuge provisioning depended strongly on predator and prey identity. Contrary to our expectations, plant structural complexity was not consistently a major driver in shaping refuge provisioning. In some cases, the level of protection depended on species-specific interactions between plant species, prey species, and plant density. Interestingly, our results on gammarid survival under carp predation were clearly different for artificial and living plants.

### Native versus non-native plants

Invading plants typically lower native fish, plant, and macroinvertebrate species abundance [32, 35] and modify the habitat structure of ecosystems [4]. Consequentially, the change in habitat structure caused by replacement of native by non-native plants could change species assemblages by altering predator-prey dynamics [10–12, 15, 39]. Contrary to our expectations, native and non-native plants functioned similarly in terms of refuge provision to macroinvertebrate prey in our experiments. Native and non-native plants in our study spanned a similar range in fractal dimensions, which may explain why they did not differ in their provision of refuge to invertebrate prey. There were specific exceptions with regard to *Gammarus pulex* prey however, as the native plant *Ceratophyllum demersum* provided refuge under carp predation, while the non-native species *Cabomba caroliniana* in low density made *G*. *pulex* more susceptible to dragonfly larvae predation.

Species-specificity is also reported by other authors. Some threatened macroinvertebrates and fish species depend on plant species for refuge, such as the *C*. *demersum* or *Stratiotes aloides* [5]. Their replacement by non-native plant structures could thus threaten conservation efforts. For example, ponds invaded by floating invasive plant species had reduced macroinvertebrate abundance and lacked sensitive benthic species like mayflies, compared to uninvaded ponds dominated by submerged native plants [32]. These species-specific effects show the utility of testing multiple plant species in experiments, both to uncover species-specific effects, and in order to generalize conclusions [52]. In future experiments, it would be of interest to assess the potential benefit of rare plant species in refuge provisioning on top of that already provided by dominant plants.

### Prey identity

Prey identity can affect predator-prey dynamics [9, 53, 54]. We show that the use of plant structures for shelter by macroinvertebrate prey under carp predation differs and conclude that plant refuge is prey-specific. Surprisingly, the presence of plants did not affect *Daphnia pulex* survival under carp predation, even though such an effect has been reported in literature [30, 55]. In the presence of predatory fish, daphnids have been observed to seek refuge in plants [56]. Yet in our trials *D*. *pulex* individuals evaded plants, a behavioural observation that has also been reported previously in lab experiments [57, 58]. It seems that *D*. *pulex* failed to detect, or respond to, the fish as it may have done in prior studies [53, 56]. The absence of anti-predator behaviour can indicate that the daphnids used had not been adapted to fish predation [59].

Contrary to the pelagic *D*. *pulex*, phytophilic clasping damselfly larvae attach themselves to a leaf or stem and rely on crypsis to survive [60]. Upon release in the experimental arena for pre-trial acclimation, damselfly larvae quickly settled and rarely moved thereafter. All plant species, including those of low complexity, provided concealment for this prey. The first larval prey consumed by carp had often not settled on plants, but was attached to the glass. This indicates that edge effects are present in aquarium experiments, and that *in situ*, efficient crypsis relies on adequate prey behaviour in seeking suitable structures to attach to. Furthermore, edge effects can inflate experimental predation rates compared to natural predation rates, because in aquarium experiments, prey cannot escape predation by moving to other areas. At high plant density, larval survival was lower, possibly because damselfly larvae positioned themselves at the outer bounds of dense plant patches where they were more exposed to carp (pers. observation). Presumably damselfly larvae did so because they perceived the outer bounds as safer habitat than the inside of vegetation. The perception of predation risk by prey has previously been shown to strongly affect prey behaviour [24]. Specifically, juvenile roach (*Rutilus rutilus*) respond adaptively to olfactory and visual cues of open-water versus ambush predators and thereby increase its chance of survival. In our study, although there was neither ambush predator nor an olfactory cue, damselfly larvae chose not to enter dense vegetation. This behaviour occurred despite the visible presence of carp in the open water.

We observed a plant species effect for the highly active benthic amphipod *Gammarus pulex* as only *Ceratophyllum demersum* provided refuge. Important for the provision of predator-free space is the body size of predator and prey in relation to interstitial plant space [29, 61]. This plant’s dense and rigid leaf whorls offered refuge to the small and agile *G*. *pulex* whenever a carp tried to hunt it down, so that *C*. *demersum* effectively provided predator-free space.

We also hypothesized that prey survival would be positively related to plant complexity expressed as the fractal dimension [37]. Surprisingly, while the fractal dimension differed among plant species, it was only positively related to damselfly larvae survival in plant patches of high density. This could indicate an effect threshold for complexity similar to threshold effects reported for density [18, 62]. However to test this idea, further experiments are required. We suggest that predator and prey identity overruled the impact of plant structural complexity and shoot density in refuge provision, at least in the range of variation in complexity and stem density used in our experiments.

### Artificial plant refuge

In contrast to our results with real plants, multiple studies using artificial plants showed that increased plant structural complexity generally improves prey survival under fish predation [18, 21], whereas there was not always an effect of stem density [6]. Such enhanced survival has been related to either concealment and reduced encounters [8, 18] or to provisioning of predator-free space [5, 17]. Similarly, we found that when using artificial plants, gammarid survival under carp predation increased with increasing qualitative structural complexity, not fractal dimension, and shoot density. Predator-free space (and consequently prey survival) is larger in artificial plants of high complexity. Plants with dense leaf whorls effectively limit fish movement, whereas plants with sparse leaf whorls like the *Elodea* analogue, or those with singular leaves like the *Vallisneria* analogue provided some predator-free habitat, but required high shoot densities to do so. However, gammarid survival was not related to the fractal dimension of the artificial plants, suggesting that the fractal dimension may not adequately capture the refuge provisioning of these plants. This may be related to the scale at which fractal dimension is estimated, *i*.*e*. at whole shoot or leaf, and it may be difficult to integrate both measures into a single index. This and other limitations of the fractal dimension, such as failing to capture diversity of complexity and size elements, have been previously discussed in a recent review [63].

### Plant rigidity

Real and artificial plants were similar in size, shape, and structural complexity, suggesting that they could have offered the same degree of concealment to prey, yet they did not. Therefore, it is interesting to note that our plants varied in rigidity, which is common for aquatic plants [64] due to trade-offs in energy expenditure, light capture, and water velocity [65]. Interestingly, a recent study measured artificial plant stiffness and showed that increased shoot stiffness slightly decreased newt foraging rates on damselfly larvae [66]. Although we did not measure plant rigidity (*e*.*g*. using Young’s modulus or stiffness), it seemed that articifial plants and *C*. *demersum* were more rigid than all other real plants. If held outside water, the shoots and branches of our artificial plants and living *C*. *demersum* retained their shape better than real plants (S2 Fig). Rigidity is therefore the most likely factor explaining the differences in refuge provisioning, which matches with our observations of fish not entering dense patches of artificial plants. Therefore, the role of structural complexity might be context-dependent [67]: plant density and complexity only become functional if plant rigidity is sufficiently high. This is reflected by robust leaf axils of *Stratiotes aloides*, root mats of floating plants, and emergent reeds providing effective physical refuge to prey [5, 68, 69]. We suggest that plant rigidity could be an important plant trait influencing predatory-prey dynamics.

### Predator hunting mode

Predator identity matters for predator-prey dynamics [40, 70, 71]. We hypothesized that predator identity, especially their hunting mode, would be an important variable that determines whether plants offer protection to prey [9, 22]. Indeed, the role of plants as refuge to *G*. *pulex* differed between both predators. Under carp predation only *C*. *demersum* provided refuge to *G*. *pulex*, whereas in the predation trials with *A*. *imperator* larvae all plant species increased *G*. *pulex* survival. This highlights the importance of studying different predators or evaluating different predator tactics when estimating refuge effects for macroinvertebrates [9]. It again provides an example of context-dependency when elucidating the functional importance of plant traits. Dragonfly larvae excel at ambushing prey from the concealment of plants by using retractable mouthparts to catch their prey [60]. As gammarids were highly active, frequently entering plant patches, their survival likely depended on chance encounter rates and the foraging efficiency of the dragonfly larvae. A previous study also showed that vegetation provides benthic prey with refuge if under predation by phytophilic predators [9]. This increased refuge to benthic prey results from vegetation restricting predator vision and movement. By residing in vegetation, dragonfly larvae predators sacrifice foraging speed for increased safety against fish predation [72]. In habitats with both actively hunting and ambushing predators, plants can simultaneously be a site of refuge and danger [73] depending largely on fauna microhabitat use [9].

In laboratory experiments, predator behaviour can be affected by stress and learning. We attempted to limit fish stress by using swim-through hatches instead of nets to move fish to the experimental arena, and by letting this schooling fish species forage in pairs. Another important aspect of predator-prey interactions is learning. Bluegill sunfish learn that searching for prey more slowly in vegetation improves foraging efficiency [74] and without vegetation this efficiency can be upped fourfold by improving handling and search time [75]. As carp are better learners than bluegill sunfish [76], it is likely that over time, the repeated use of carp increased their foraging efficiency as would happen in natural environments. Due to the randomized testing of plant species across fish pairs over time, the prey survival reported is the averaged efficiency of fish at various stages of experience. Carp learning and their confinement to the experimental arena resulted in a high predation pressure. Whereas in natural systems fish might evade non-profitable habitat such as vegetated areas and find easier prey in open water [75, 77], here carp had to forage in vegetated habitats. This they did successfully, as only prey well hidden in interstitial space inaccessible to carp survived. Compared to the efficient carp, dragonfly larvae foraging was slower and less reliant on searching. Therefore, refuge requirements for prey to benefit under ambushing dragonfly predation appear less demanding than those under searching carp predation.

### Synthesis

In conclusion, native and non-native submerged aquatic plants functioned similarly in terms of refuge provision. Instead of plant origin, refuge provision was largely determined by predator and prey identity and only weakly by plant complexity or shoot density. Interestingly, our study indicated that plant rigidity, which is higher for artificial plants than for their corresponding living counterparts, might be a major trait in refuge provisioning. The role of plant complexity in refuge provision is noticeable only when plants are sufficiently rigid. Therefore, results on predator-prey dynamics obtained using artificial plant analogues should be interpreted with care when extrapolating to the effects of living plants. Altogether, these results imply that modification of predator-prey interactions through plant invasions, if present, may occur only when non-native plant species are of strongly contrasting growth form, morphology, rigidity, and density compared to the native plant species.

## Supporting Information

S1 Fig Close-up of an experimental arena. Photograph of an experimental area planted with *Myriophyllum spicatum* in low density, while on the left a wooden wall separates the fish living area and the experimental area in view. The lower part of this separator acted as a hatch which allowed fish to swim into the experimental area on their own, thereby reducing stress.
(TIF)

S2 Fig Qualitative impression of the rigidity of artificial and real aquatic plants. Four artificial plant analogues and two real plants were attached horizontally to a vertical metal bar and photographed. The rigidity of whole shoots (longitudinal axis) and branches (lateral axis) is classified as rigid (check mark), less rigid (cross), or not available (NA).
(TIF)

S3 Fig Fractal dimension versus macroinvertebrate prey survival correlation plots. Mean *Daphnia pulex* (circles), *Gammarus pulex* (squares with refuge of living plants and downward triangles with refuge of artificial plants) and damselfly larvae (upward triangles) survival under mirror carp predation in plant refuge of low (closed symbols) or high plant density (open symbols) plotted against the cross-sectional (**A**) and shoot fractal dimension (**B**) of plants. Only significant regression lines were plotted for graphical clarity.
(TIF)

S1 Table Water quality data of carp predation trials. Mean ± SEM value of multiple parameters over time (n = 24 for water characteristics, n = 5 for nutrient data).
(DOCX)

S2 Table Mirror carp pre- and post-experiment information for each individual. (DOCX)
