# Supplementary figures and images for: Native and Non-Native Plants Provide Similar Refuge to Invertebrate Prey, but Less than Artificial Plants
Source: PLoS One. 2015 Apr 17;10(4):e0124455. doi: 10.1371/journal.pone.0124455 (PMC4401678; doi:10.1371/journal.pone.0124455)

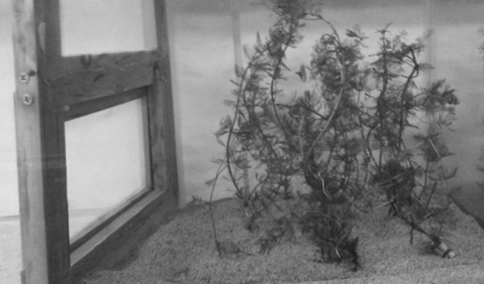

Supplement: S1 Fig — Photograph of an experimental area planted with Myriophyllum spicatum in low density, while on the left a wooden wall separates the fish living area and the experimental area in view. The lower part of this separator acted as a hatch which allowed fish to swim into the experimental area on their own, thereby reducing stress. (TIF) [file pone.0124455.s001.tif]

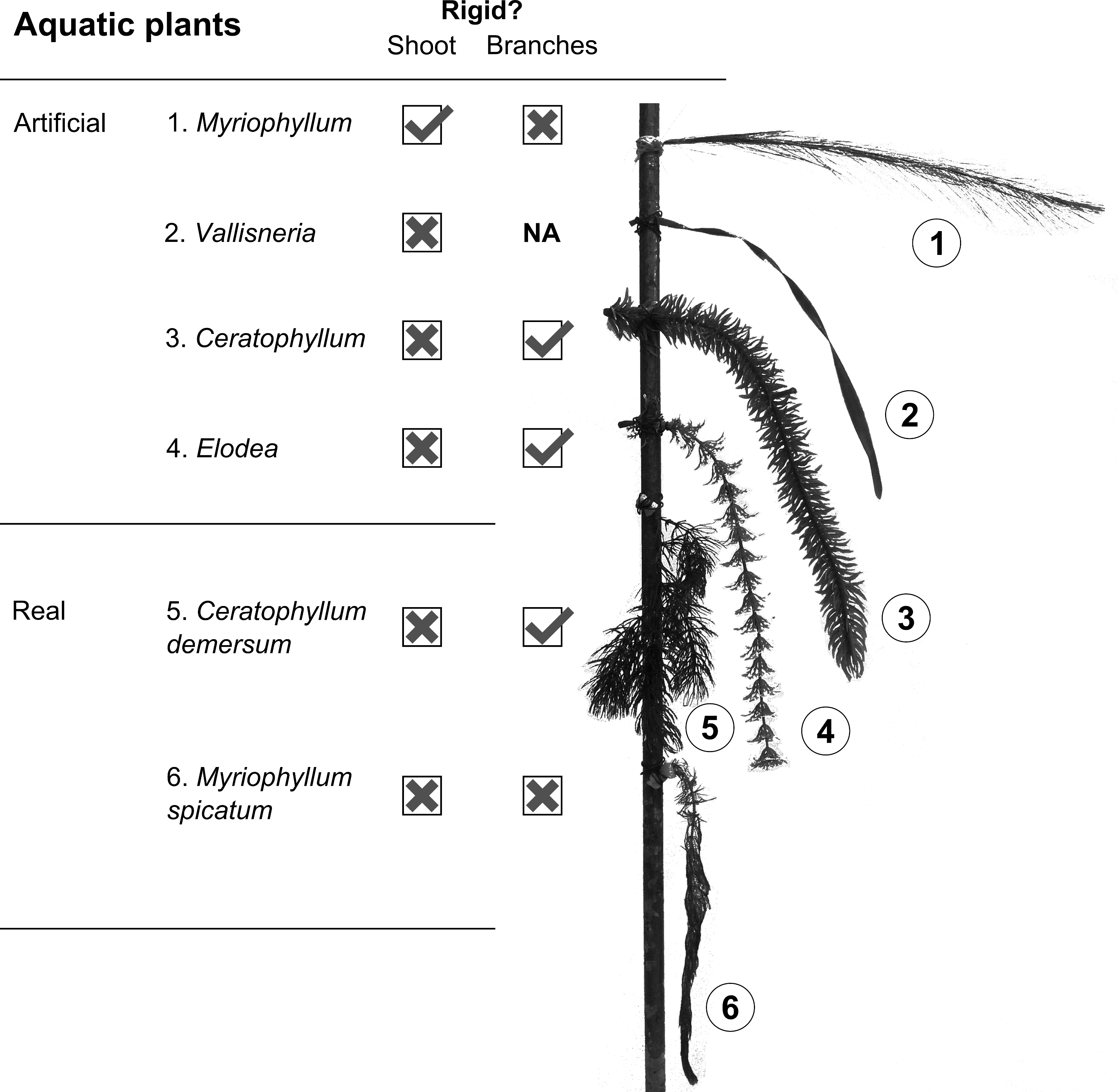

Supplement: S2 Fig — Four artificial plant analogues and two real plants were attached horizontally to a vertical metal bar and photographed. The rigidity of whole shoots (longitudinal axis) and branches (lateral axis) is classified as rigid (check mark), less rigid (cross), or not available (NA). (TIF) [file pone.0124455.s002.tif]

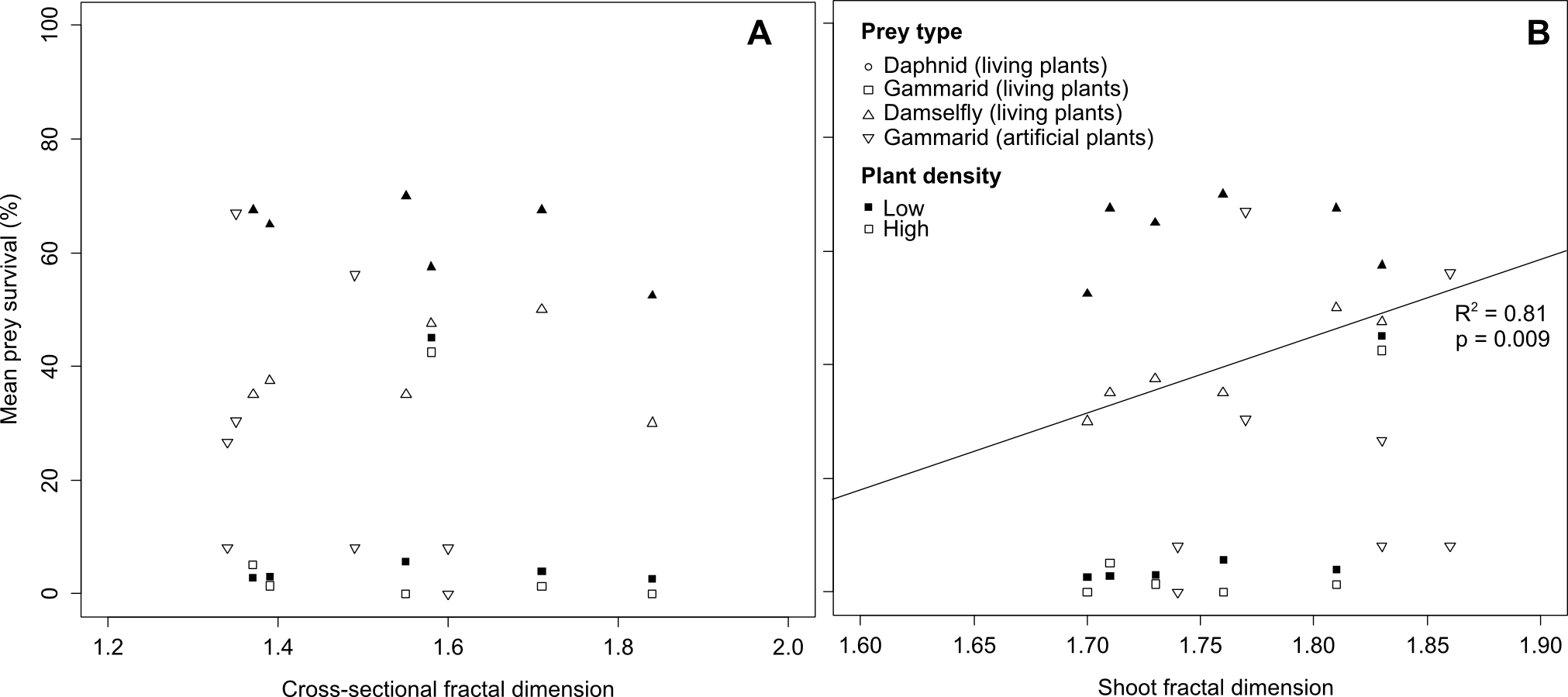

Supplement: S3 Fig — Mean Daphnia pulex (circles), Gammarus pulex (squares with refuge of living plants and downward triangles with refuge of artificial plants) and damselfly larvae (upward triangles) survival under mirror carp predation in plant refuge of low (closed symbols) or high plant density (open symbols) plotted against the cross-sectional (A) and shoot fractal dimension (B) of plants. Only significant regression lines were plotted for graphical clarity. (TIF) [file pone.0124455.s003.tif]
