# Supplementary material for: Native and Non-Native Plants Provide Similar Refuge to Invertebrate Prey, but Less than Artificial Plants
Source: PLoS One. 2015 Apr 17;10(4):e0124455. doi: 10.1371/journal.pone.0124455 (PMC4401678; doi:10.1371/journal.pone.0124455)
Supplement: S1 Table — Mean ± SEM value of multiple parameters over time (n = 24 for water characteristics, n = 5 for nutrient data). (DOCX) [file pone.0124455.s004.docx]

Table S1 **Water quality in the aquaria used for the mirror carp predation trials displaying the mean ± SEM value of multiple parameters over time (n = 24 for water characteristics, n = 5 for nutrient data)**

| Aquarium | pH | Conductivity (µS/cm) | Oxygen (mg/L) | Temperature (°C) | Nitrate (mg/L) | Nitrite (µg/L) | Ammonium (µg/L) |
| --- | --- | --- | --- | --- | --- | --- | --- |
| 1 | 7.6 ± 0.1 | 291 ± 18 | 7.6 ± 0.3 | 21.0 ± 0.5 | 3.1 ± 1.0 | 5.6 ± 1.5 | 12.8 ± 3.0 |
| 2 | 7.7 ± 0.1 | 291 ± 17 | 7.6 ± 0.4 | 21.0 ± 0.5 | 3.1 ± 0.6 | 5.8 ± 0.8 | 13.0 ± 2.2 |
| 3 | 7.7 ± 0.1 | 286 ± 19 | 7.6 ± 0.4 | 20.9 ± 0.5 | 3.0 ± 1.1 | 5.0 ± 1.6 | 18.4 ± 15.3 |
| 4 | 7.7 ± 0.1 | 286 ± 19 | 7.5 ± 0.4 | 20.8 ± 0.5 | 2.8 ± 0.6 | 5.0 ± 0.7 | 14.8 ± 7.3 |
| 5 | 7.7 ± 0.1 | 271 ± 11 | 7.6 ± 0.4 | 20.8 ± 0.5 | 1.9 ± 0.5 | 4.8 ± 1.3 | 13.8 ± 2.5 |
| 6 | 7.7 ± 0.1 | 271 ± 11 | 7.6 ± 0.3 | 20.8 ± 0.5 | 1.7 ± 0.4 | 4.6 ± 0.9 | 10.0 ± 3.2 |
| 7 | 7.7 ± 0.1 | 264 ± 12 | 7.7 ± 0.3 | 20.7 ± 0.5 | 2.0 ± 0.6 | 4.8 ± 0.8 | 11.6 ± 3.7 |
| 8 | 7.8 ± 0.1 | 263 ± 12 | 7.7 ± 0.4 | 20.6 ± 0.5 | 2.2 ± 0.5 | 4.8 ± 0.8 | 12.8 ± 6.8 |
