# Supplementary material for: Native and Non-Native Plants Provide Similar Refuge to Invertebrate Prey, but Less than Artificial Plants
Source: PLoS One. 2015 Apr 17;10(4):e0124455. doi: 10.1371/journal.pone.0124455 (PMC4401678; doi:10.1371/journal.pone.0124455)
Supplement: S2 Table — (DOCX) [file pone.0124455.s005.docx]

Table S2 **Mirror carp parameters measured at the start and end of the experiment given for each individual along with its assigned aquarium**

|  | | Pre experiment |  | Post experiment | | |
| --- | --- | --- | --- | --- | --- | --- |
|  |  |  |  |  |  |  |
| Aquarium | Fish | Wet weight (g) |  | Standard Length (mm) | Wet weight (g) | Gape width (mm) |
| 1 | A | 9.5 |  | 72 | 12.2 | 7.0 |
|  | B | 11.2 |  | 78 | 15.3 | 8.2 |
| 2 | A | 9.9 |  | 74 | 13.2 | 7.4 |
|  | B | 10.4 |  | 79 | 15.4 | 7.8 |
| 3 | A | 8.8 |  | 74 | 12.9 | 7.8 |
|  | B | 11.8 |  | 76 | 13.9 | 7.9 |
| 4 | A | 11.6 |  | 77 | 13.9 | 6.9 |
|  | B | 9.0 |  | 75 | 12.6 | 7.0 |
| 5 | A | 11.3 |  | 76 | 14.7 | 7.9 |
|  | B | 9.4 |  | 75 | 13.4 | 7.6 |
| 6 | A | 8.6 |  | 75 | 13.4 | 7.4 |
|  | B | 12.0 |  | 78 | 16.1 | 8.0 |
| 7 | A | 10.9 |  | 79 | 15.8 | 8.1 |
|  | B | 9.8 |  | 76 | 13.7 | 7.4 |
| 8 | A | 12.3 |  | 82 | 17.4 | 7.9 |
|  | B | 8.3 |  | 75 | 14.7 | 7.4 |
